# A Multi-Stage Visible and Infrared Image Fusion Network Based on Attention Mechanism

**DOI:** 10.3390/s22103651

**Published:** 2022-05-11

**Authors:** Xin Zheng, Qiyong Yang, Pengbo Si, Qiang Wu

**Affiliations:** Faculty of Information Technology, Beijing University of Technology, Beijing 100124, China; qyyang1997@163.com (Q.Y.); sipengbo@bjut.edu.cn (P.S.); qiangwu@bjut.edu.cn (Q.W.)

**Keywords:** deep learning, image fusion, attention mechanism

## Abstract

Pixel-level image fusion is an effective way to fully exploit the rich texture information of visible images and the salient target characteristics of infrared images. With the development of deep learning technology in recent years, the image fusion algorithm based on this method has also achieved great success. However, owing to the lack of sufficient and reliable paired data and a nonexistent ideal fusion result as supervision, it is difficult to design a precise network training mode. Moreover, the manual fusion strategy has difficulty ensuring the full use of information, which easily causes redundancy and omittance. To solve the above problems, this paper proposes a multi-stage visible and infrared image fusion network based on an attention mechanism (MSFAM). Our method stabilizes the training process through multi-stage training and enhances features by the learning attention fusion block. To improve the network effect, we further design a Semantic Constraint module and Push–Pull loss function for the fusion task. Compared with several recently used methods, the qualitative comparison intuitively shows more beautiful and natural fusion results by our model with a stronger applicability. For quantitative experiments, MSFAM achieves the best results in three of the six frequently used metrics in fusion tasks, while other methods only obtain good scores on a single metric or a few metrics. Besides, a commonly used high-level semantic task, i.e., object detection, is used to prove its greater benefits for downstream tasks compared with single-light images and fusion results by existing methods. All these experiments prove the superiority and effectiveness of our algorithm.

## 1. Introduction

Image fusion outputs a single image with rich information through the complementarity between multiple images of different illumination [1], sensors [2], and focuses [3] in the same scene. Among them, one of the most widely studied tasks is the image fusion of different sensors, especially visible light and infrared [4]. Owing to the difference in acquisition bands, each type of image has significant differences in characteristics. Visible image acquisition through light reflection obtains more abundant detailed texture information but less common contrast. An infrared image shows the temperature of the object with obvious global contrast with only the approximate edge contour of the object [5]. There is a certain complementarity between the two types of images. Through fusion, several targeted algorithms can effectively combine the advantages of both and retain the detailed texture and significant target characteristics in the same image.

The current fusion algorithm, whether based on the traditional algorithm or deep learning, primarily contains parts of image feature extraction and multi-feature fusion and image reconstruction. Among these, the traditional fusion algorithm realizes the entire algorithm flow through manual design, which can be roughly divided into sparse representation-based [3], multi-scale transform-based [6], and saliency-based design [7]. Deep learning algorithms replace some or all of the progress with a neural network. According to the difference of training methods, this paper divides them into three modes: “no training”, “reconstruction training”, and “fusion training”, the last two of which are the main focus of this paper.

**Figure 1:**
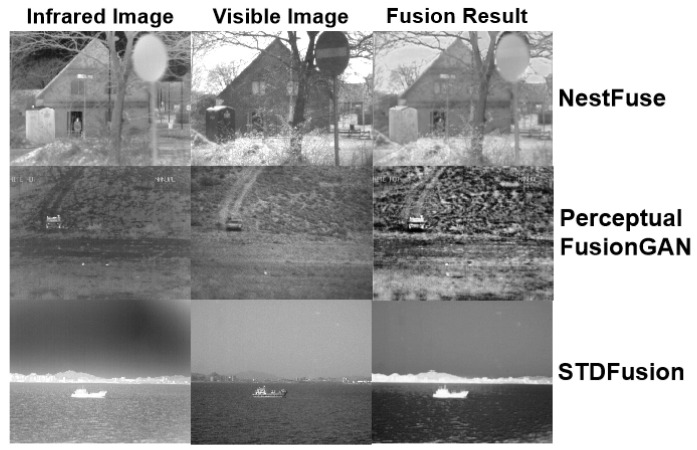
Visualized results of some existing fusion methods in typical images.

Although existing algorithms have achieved certain results, they are not ideal in specific scenarios. The “reconstruction training” method reduces the training difficulty in a fake supervised way. However, its manually designed dual-light fusion module easily causes underutilization of features owing to its incompatibility with deep neural networks. NestFuse [8] is a recently published method that uses a hand-designed attention fusion module to combine dual-light features. However, owing to the weighted average calculation, the fusion function is much more inclined toward high-amplitude features, like a brighter region, which may result in undesirable feature filtering. This problem is shown in the first line of Figure 1, where the high intensity of the sign region in an infrared image suppresses the corresponding visible area, causing NestFuse to lose the visible texture structure information. The other type of mode, called “fusion training”, uses an end-to-end network to complete the entire fusion task, with a fuller utilization of features but greater difficulty in unsupervised training. In particular, when using Generative Adversarial Network (GAN) architecture on a small sample training set, artifacts and unnatural phenomena occur in the fusion result [9]. In the middle of Figure 1, the fusion result output by Perceptual FusionGAN [10] shows an unnatural grayscale distribution with a certain amount of streak noise, which affects the overall fusion effect seriously. In other ways, owing to the unsupervised training pattern of the fusion task, the loss function completely dominates the training process of the model. However, the current existing loss function simply relies on the L1 and L2 loss design of the intensity and gradient, and the weight ratio relies on the human setting; this cannot ensure that the network learns a truly reasonable fusion strategy, resulting in a biased fused image or an improper grayscale distribution. To highlight the target, STDFusion [11] sets the different ratios of pixels and gradient loss for the foreground and background area by a pre-labeled mask. However, as shown in the last line of Figure 1, the manual hyperparameter is not appropriate for all the scenarios, which may cause excessive highlighting of the target; this results in less detailed information, which makes the fused image close to the binary image as a whole.

To solve the above problems, in this paper, a multi-stage visible and infrared image fusion network based on the attention mechanism (MSFAM) is proposed that mainly consists of two aspects: model structure and training strategy. For the aspect of model structure, the “reconstruction training” and “fusion training” modes have their advantages. Existing algorithms do not integrate them, which leaves potential room for improvement. The relative independence of the two modes mainly stems from the mismatched dimensions of the input feature of the reconstruction module. Therefore, the primary motivation of this paper is to design a fusion model that can satisfy the requirements of multi-stage training and achieve a combination of advantages to enhance the final fusion effect. To further improve the accuracy of feature utilization, a well-designed attention mechanism was integrated in our fusion model. In addition, for the training strategy, we addressed the difficulty of unsupervised training from two different perspectives. First, without ideal image labels, the network cannot learn the data distribution of the real fusion, leading to instability in learning. Fortunately, neural networks can be used to fit the data distribution to a certain extent. GANMcC [12] uses a discriminator in the GAN to predict the input image data characteristics, constraining the output to meet both visible and infrared data distribution simultaneously. Considering the instability of GAN model training, we adopted a pre-trained CNN module based on the idea of deep semantic constraint, which use a specific high-level task to assist the training of low-level image processes. Second, aiming at the constant bias and inaccurate fusion description caused by manual setting loss, we designed a loss function called “Pull–Push Loss” that automatically adjusts the weights to fine-tune the training trend of the model according to the current learning status, ensure its training balance, and fully learn the dual-source information. By combining the above design, the MSFAM algorithm can output fused images conforming to human eye standards or a quantitative index with visible detail information and infrared salient features.

The main contributions of this paper are as follows:

1. We present a multi-stage training pattern of the fusion model, as well as a feature fusion module based on an attention mechanism to prove its feasibility. The model combines the advantages of the two existing training methods, which effectively improves the quality of fused images.
2. To improve the amount of information contained in the output results while reducing the difficulty in model training, we designed a Semantic Constraint module and Pull–Push loss function for training assistance.
3. By combining the above methods, the proposed MSFAM achieves a better performance in several evaluation metrics of public datasets, including TNO [13] and RoadScene [14,15]; we hope it can provide a novel pattern for future image fusion tasks.

The rest of this paper is organized as follows: Section 2 presents some of the research background related to this paper. In Section 3, we detail the proposed MSFAM model. In Section 4, we illustrate the experimental details related to the model, including an ablation study and comparisons. Section 5 provides the conclusions and outlook for the work.

## 2. Related Work

In this section, we provide an overview of the literature relevant to this paper. We first outline the existing deep-learning-based infrared and visible image fusion algorithms to illustrate its recent development. In the following sections, several related ideas and techniques for the attention fusion module and Semantic Constraint module are described.

### 2.1. Deep-Learning-Based Image Fusion Methods

We analyzed the existing deep-learning-based fusion algorithms and classified them into three categories according to the training mode: “no training”, “reconstruction training”, and “fusion training”.

The “no training” mode only uses a pre-trained CNN, such as Resnet [16] or Densenet [17], as a generic feature extractor by removing the final fully connected layer. Afterward, the final fused image is obtained by processing the original images with a weight map generated by a hand-designed computation method on the neural network features.

Based on CNNs used for feature extraction, the “reconstruction training” model leaves the reconstruction task to the network similarly. It uses two different computational approaches in the training and prediction phases. During training, the network only includes the feature extraction part and the image reconstruction part [8,18,19,20]. Through the reconstruction training of single-light images, the model is given the ability of feature extraction and reconstruction. In the prediction stage, the human-designed fusion module is used to achieve effective fusion of dual-light features. It ensures that the input feature dimensions of the reconstruction part are the same as in the training phase so that the trained reconstruction module can be used to output fused results.

Compared to the above two types of approaches, “fusion training” is the most intuitive fusion mode, in which a well-designed end-to-end fusion model outputs fusion results directly from the input dual-light images. In contrast to the “reconstruction training” mode, the feature fusion part is usually designed as dual-light feature concatenation, and the interaction is implicitly implemented in the subsequent image reconstruction process [11,14,21]. In addition, for the GAN commonly used in this mode, the fusion model is always trained simultaneously with the designed discriminators [10,12,22,23,24,25]. In the process of adversarial gaming, it simultaneously improves the respective capabilities of both sides to achieve the improvement of the fusion effect.

However, the inconsistent input feature dimension of the reconstruction module causes a gap in the “reconstruction training” and “fusion training” mode. To combine the advantages of each, a multi-stage fusion approach with a learning fusion module was designed for this paper, which results in a visible and infrared fused image with rich structural information and significant target grayscale.

### 2.2. Attention Mechanism

Owing to the bottlenecks in information processing, humans selectively focus on a portion of all information while ignoring the rest of it [26]. The above behavior is caused by the attention mechanism. In recent years, many researchers have realized the importance of the attention mechanism. It has been studied in various aspects, and some researchers have tried to introduce it into neural networks to make them more biased toward effective information to enhance the overall effectiveness of their models [27].

SEnet [28] introduced a channel attention mechanism by achieving the channel-by-channel weighted vector of features. It enables the network to focus more on the effective channel and achieve adaptive feature enhancement and decay. Accordingly, CBAM [29] includes a similar attention mechanism from the spatial location perspective, empowering networks to focus on the key locations. In addition, multiple attention extraction methods enable a more accurate representation of the importance, which results in a better effect. The authors of [30,31] introduced the attention mechanism into a sea-surface ship detection task and achieved better results by extracting more accurate features.

However, as the convolution is only computed in the adjacent regions, which causes the loss of some large span associations, non-local neural networks [32] and transformers [33] achieve the interaction of features between different locations by linear operations to capture the global information and ensure the accuracy of attention calculation.

We designed the attention feature fusion module based on the CBAM module to enhance the fusion effect. This module uses the attention mechanism to achieve adaptive fusion of infrared features and visible features, avoiding information loss and redundancy. Compared with the manually designed method, the learning feature fusion function can better meet the CNN reconstruction requirements and obtain favorable fusion results.

### 2.3. Semantic Constraint Method

Low-level image processing tasks, such as image denoising and defogging, mainly aim to serve high-level tasks, such as pre-processing, to improve classification or detection performance. In recent years, researchers have found that the training result of low-level tasks can be effectively improved with the assistance of high-level networks, which is called semantic constraint.

Liu [34] and Wang [35] jointly trained the denoising model by connecting multiple frozen high-level networks at the back end of it, similar to the AODNet [36], using a well-trained Faster RCNN detection model, effectively improving the visual effect of the output image. TBCNet [37] uses an image segmentation model to achieve saliency extraction for weak targets, which can still be regarded as a pixel-level low-level task. Therefore, the detection effect is improved by adding a classification model at the back end for predicting the number of targets in the saliency map, constraining the segmentation output to avoid false detection caused by noise.

The image fusion task studied in this paper also belongs to the field of low-level image processing, but it is difficult to train a high-level detection or segmentation network due to the lack of data. Therefore, we designed a novel Semantic Constraint module using multi-label classification to predict the degree of conformity between the fused image and the real visible and infrared image data distribution. The training result of the fusion model is effectively improved through the continuous increase of the information content contained in the fused image from dual-source images.

## 3. Approach

This section specifies our proposed MSFAM model. First, we give an overview of the proposed method in Section 3.1; in Section 3.2, Section 3.3, Section 3.4, Section 3.5 and Section 3.6, we elaborate on the key points of MSFAM as follows: the overview and details of the network, the multi-stage training approach, the attention fusion part, the Semantic Constraint module, and the Pull–Push loss function.

### 3.1. Overview

**Figure 2:**
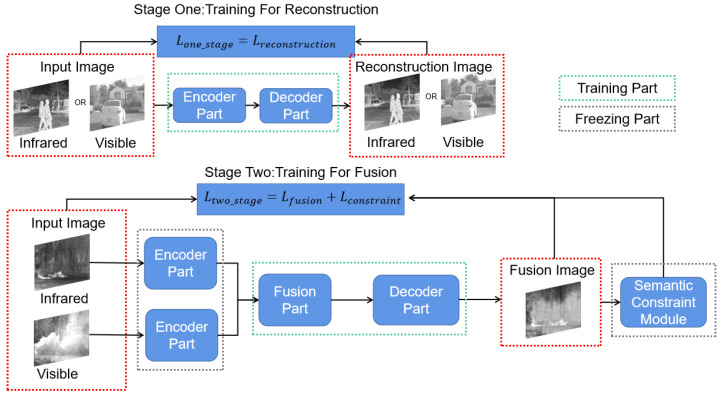
An illustration of our multi-stage training approach, which contains multiple training stages, with the encoder, decoder, and feature fusion parts (involved in the second stage).

Based on the above observations, we propose an image fusion network with multiple training stages to achieve the effective combination of visible texture information and infrared salient features (Figure 2). Specifically, the multi-stage training mode effectively avoids the difficulty of scratch training based on the first-stage training. Through the second stage, the network—especially the feature fusion module—learns to pay more attention to the effective dual-light features. To provide feasibility for multi-stage training, we specially designed a feature fusion module based on the attention mechanism. It realizes effective attention through the interaction and enhancement of dual-light features. In addition, to further improve the result of unsupervised training, the Semantic Constraint module and Pull–Push loss function were designed for the training stage. The Semantic Constraint module achieves the fitting of infrared and visible data distribution by a multi-label classification network, which enables the fusion result to satisfy each distribution directly, ensuring that the output contains as much valid information from both images as possible. However, the Pull–Push loss forces the output results to oscillate between infrared and visible images by an adaptive loss weight to extend the effective training so that it avoids the overfitting caused by small-scale data sets.

### 3.2. Network Structure

**Figure 3:**
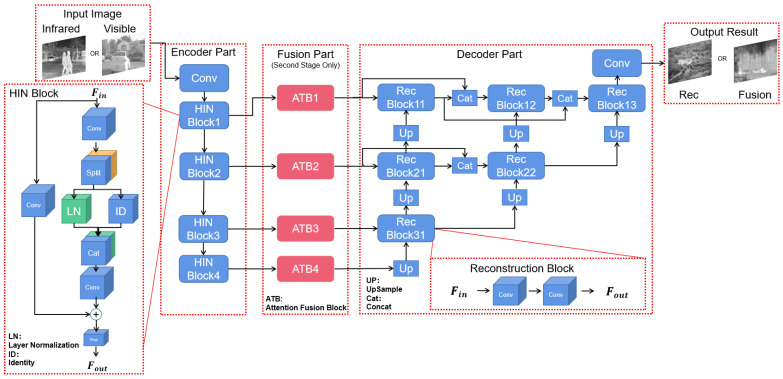
The detail structure of the proposed multi-stage fusion network.

The overall structure of our MSFAM model is shown in Figure 3. Considering the superiority of the NestFuse [8] fusion effect, the Unet++ structure of its backbone was also adopted in this paper. Through multiple consecutive encoding modules, the network achieves feature extraction at different scales and levels. Subsequently, multiple decoding modules are used to realize the image reconstruction work by combining the near skip-connected operation for the reuse of the earlier feature maps. Among them, the near skip-connected operation promotes the retention of effective information and improves the detail effect of the overall fused image while avoiding the unexpected result caused by cross-stage cascading.

The original encoding module uses successive Conv layers to achieve feature extraction of the image, and this straight structure is prone to the loss of early texture detail information owing to the non-use of the Resnet block structure. In addition, owing to the proposed multi-stage training method, the network without a normalization layer may lead to unstable model output and reduce the convergence efficiency and effect of the network. Therefore, this paper uses HINBlock in HINet [38] instead of the original encoding module, which uses half instance normalization to avoid the performance degradation of low-level tasks, such as image denoising and reconstruction, caused by traditional normalization methods, such as Batchnorm and LayerNorm. It effectively improves the fusion effect and convergence speed of the model.

Besides the basic encoding and decoding structure, an attention fusion module was designed similar to the “fusion training” mode. However, to accommodate the multi-stage training approach proposed in this paper, the designed module is learnable with parameters. Details are described in Section 3.4.

### 3.3. Multi-Stage Training Approach

To make better use of neural networks, we focused on the “reconstruction training” and “fusion training” modes. Through the above analysis in Section 1, we can see that these two approaches have some complementarity in terms of advantages. “Reconstruction training” causes less training difficulty and “fusion training” realizes a more full utilization of information. Therefore, to effectively combine the benefits of the two modes, we designed a multi-stage fusion model training method, including both “reconstruction training” and “fusion training,” which is shown in Figure 2.

In the first “reconstruction” phase, we train the above-designed encoding and decoding part of the model with a single visible or infrared image to achieve feature extraction and image reconstruction capability. In this paper, we use L1 loss and SSIM loss to realize one-stage training, and the loss function is as follows:

(1) $$L_{one_{stage}}\left(I,O\right)=L_{1}\left(I,O\right)+\alpha_{1}L_{SSIM}\left(I,O\right)=\|I-O\|+\alpha_{1}\left(1-SSIM\left(I,O\right)\right),$$

where $\alpha_{1}$ is the scale factor of SSIM loss, and 0.84 is chosen according to [39].

Based on the preliminary training model obtained, the attention fusion module and the Semantic Constraint module are added to the model for the second-stage “fusion training”. The two-stage training loss function is as shown as Equation (2), which includes L1 loss, Semantic Constraint loss, and Pull–Push loss. The main purpose of this stage is to make the added attention fusion module effectively fuse the dual-light features through learning to ensure the effect of subsequent reconstruction of the image. Specifically, a small number of paired input images are fused through the feature extraction part, fusion part, and reconstruction part to achieve end-to-end fusion output, and the corresponding loss function was designed to achieve training.

(2) $$L_{two_{stage}}\left(I,V,F\right)=L_{1}\left(I,V,F\right)+\alpha_{2}L_{Constraint}\left(F\right)+\beta L_{Pull-Push}\left(I,V,F\right),$$

where $\alpha_{2}$ is the scale factor of Semantic Constraint loss, β is the factor of Pull–Push loss, and the specific details regarding Semantic Constraint loss and Pull–Push loss are described in Section 3.5 and Section 3.6, respectively.

To avoid the loss of the first-stage training effect, it is necessary to freeze the parameters in the second-stage fusion training, which include the encoding part and the decoding part. However, considering the difference between the fused features and the original ones, completely freezing the decoding module will affect the effect of image reconstruction. Therefore, for the decoding module, the learning rate is reduced by half instead of freezing so that the network has a certain adjustment ability to better use the fused features to achieve image reconstruction while retaining the pre-training effect.

### 3.4. Attention Fusion Block

To give feasibility to the two-stage training approach while improving the interaction effect of features as much as possible, we drew on the CBAM [29] to design the attention fusion block, which contains the channel attention module and the spatial attention module, as shown in Figure 1. First, we concatenate the visible features $\left(F_{\mathrm{vis}}\in {\mathbb{R}}^{C\times H\times W}\right)$ with the infrared features ($F_{\mathrm{ir}}\in {\mathbb{R}}^{C\times H\times W})$ for subsequent processing as joint features. For the feature $F_{\mathrm{concat}}\in {\mathbb{R}}^{2C\times H\times W}$, the channel attention module and the spatial attention module are used in turn to achieve effective information enhancement and ineffective feature suppression in different aspects.

**Figure 4:**
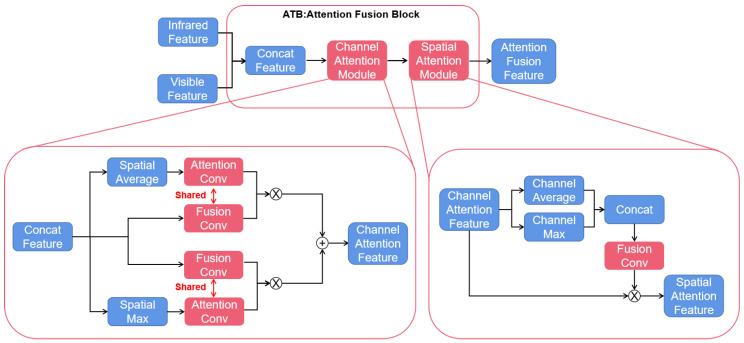
Main structure of the attention fusion block. Left part is the detailed structure of channel attention module and right part is that of spatial attention module.

The channel attention module (see left part of Figure 4) is based on CBAM with a certain difference. In the conventional CBAM, the filter and fusion of the input features are not involved, so the output feature dimension is kept the same as the input, that is, $F_{\mathrm{CA}}\in {\mathbb{R}}^{2C\times H\times W}$. In contrast, for the image fusion task in this paper, it is required that we ensure that the output features are in the same dimension as the single-light ones, that is, $F_{\mathrm{CA}}\in {\mathbb{R}}^{C\times H\times W}$, to input to the subsequent pre-training reconstruction module. Therefore, the original channel attention module is modified to obtain the feature fusion and filtering ability besides its basic attention mechanism. First, the channel of vector output by the attention Conv (CBAM uses a fully connected layer to get the attention vector, which can be replaced by a 1 × 1 Conv layer) is set to half of the input, that is, the same as that of the single-light feature, as shown in Equations (4) and (5). On this basis, a 1 × 1 fusion Conv is used to modify the dimension of the input feature. After pointwise multiply and addition, as Equation (3), the concat feature can be enhanced from channel aspect. In addition, by sharing the parameters of two Conv layers, the correlation between the attention vector and the features is achieved, ensuring the effectiveness of the attention calculation.

(3) $$F_{CA}=M_{CA-max}\left(F\right)⊗Conv_{max}^{1\times 1,2C\to C}\left(F\right)+M_{CA-avg}\left(F\right)⊗Conv_{avg}^{1\times 1,2C\to C}\left(F\right),$$

(4) $$M_{CA-max}=Sigmoid\left(Conv_{max}^{1\times 1,2C\to C}\left(MaxPool\left(F\right)\right)\right),$$

(5) $$M_{CA-avg}=Sigmoid\left(Conv_{avg}^{1\times 1,2C\to C}\left(AvgPool\left(F\right)\right)\right),$$

The spatial attention module (see right part of Figure 4) is similar to the traditional CBAM, with the adjustment of some hyperparameters. Its operations are as same as the original module, as shown as Equations (6) and (7). To better adapt to the different input scale of the four attention modules in our model, the size of the convolution kernel is modified correspondingly to 7, 5, 5, and 3 from the uniform 7 × 7, respectively, enabling the network to better understand the contextual information and obtain more accurate attention results.

(6) $$F_{SA}=M_{SA}\left(F_{CA}\right)⊗F_{CA},$$

(7) $$M_{SA}=Sigmoid\left(Conv\left(\left[AvgPool\left(F\right),MaxPool\left(F\right)\right]\right)\right),$$

In each attention module, two methods, average pooling and maximum pooling, are used to extract information in parallel and fuse the calculation results, which can avoid information omission to a certain extent and improve the overall attention quality.

### 3.5. Semantic Constraint Module

In GANMCC [12], a dual classification discriminator is used to constrain the output result, ensuring the information balance degree of the fused images. However, considering the training difficulty and instability of GAN networks, we achieved a similar aim by a pre-trained multi-label classification network. Unlike the discriminator, the network’s parameters are frozen after training, ensuring the original discriminative ability of the module without affecting the validity of the prediction result probability.

**Figure 5:**
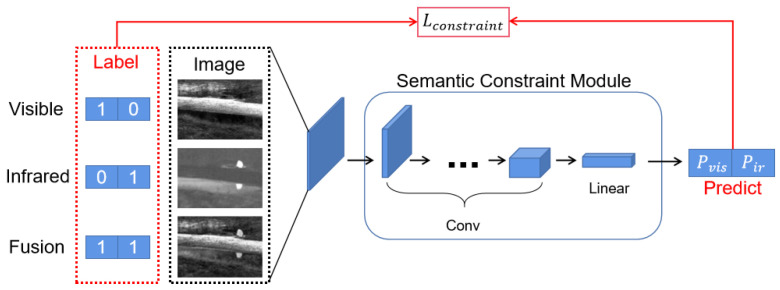
The overall workflow of the Semantic Constraint module.

The structure of the Semantic Constraint module has four Conv layers and a fully connected layer, as shown in Figure 5. Its structure is simple but effective. After training, it can achieve an accuracy of more than 99%, which is sufficient to complete the dual-light discrimination task required in this paper.

According to the task requirements, we train the constraint module as a multi-label classification model using BCE loss, which is used to determine the probability of an input image belonging to a single-light image based on its data distribution.

In the training phase, we use the original visible and infrared single-source images as part of the data, with the corresponding probability of 1 in the output label, to train the ability to discriminate the distribution difference between the dual-light data. The other part of the input uses the fusion results of some existing algorithms to train the multi-label prediction capability, whose output labels are all set to 1 for the probability of both.

In the usage phase, the fused output image is fed into the Semantic Constraint module. By optimizing corresponding loss, we can effectively ensure that the output image meets the data distribution of both visible and infrared information and increase the information of both simultaneously to achieve an effective improvement for the quality of the fusion result. The Semantic Constraint loss is as follows:

(8) $$L_{Constrain}=L_{BCE}\left(M_{Constrain}\left(F\right),label\right)=1\cdot log\left(p_{vis}\right)+1\cdot log\left(p_{ir}\right),$$

where $p_{vis},\;p_{ir}$ are the visible probability and infrared probability of the Semantic Constraint model output, respectively.

### 3.6. Pull–Push Loss Function

Considering the unsupervised task mode of the second “fusion training” stage, the design of the loss function plays a crucial role in the training effect of the model.

To prevent the training bias caused by manual weighted loss functions, we designed a novel Pull–Push loss function based on the weighted edge information evaluation index [40], combined with the widely used SSIM loss function [41]. It can determine the dual-light scaling coefficients adaptively according to the current learning status of the fusion model and realize a more flexible training process to ensure that the output results effectively fuse multi-light information without information omission.

Concretely, when the fused image is more similar to the visible one, its information preserving value is relatively high, which leads to an increase of visible loss in the loss function after weighting. To reduce the total loss, the fused model is more focused on the visible part, which will push away from visible information and move closer to infrared information during optimization. When the fusion image has a high similarity to the infrared information, it will pull the fusion image to the visible light and away from the infrared information. In the continuous Pull–Push process, the network can keep continuously learning to achieve full fusion of dual-source images.

The weighted edge information can measure the preserving degree of edge structure information in the fused image, which is used to indicate the similarity between the current fusion result and each source image in this paper. The specific calculation is shown in Algorithm 1. After obtaining the weighted edge information preserving value of the fused image with visible and infrared respectively, the final Pull–Push loss is obtained by weighting the SSIM loss function jointly through normalization, as shown in Algorithm 2. As can be seen, we use the weighted edge information preserving value to calculate the visible and infrared loss weights in the Push–Pull loss. To avoid large numerical oscillations, which lead to unstable network training, we use the Softmax operation to normalize the weights to reduce their disparity.
**Algorithm 1** Compute Weighted Edge Information Preserving Value**Input:** Single source image $I_{a}$; fusion result of MSFAM $I_{F}$**Output:** Edge information preserving value $Q^{\mathrm{ab}}$; Edge intensity weight $w_{a}$1: Calculate the x and y direction gradients of each image:$s_{a}^{x}={\mathrm{Sobel}}_{x}\left(I_{a}\right)$ $s_{a}^{y}={\mathrm{Sobel}}_{y}\left(I_{a}\right)$$s_{b}^{x}={\mathrm{Sobel}}_{x}\left(I_{b}\right)$ $s_{b}^{y}={\mathrm{Sobel}}_{y}\left(I_{b}\right)$2: Calculate the edge intensity and direction for each image:$g_{a}=\sqrt{s_{a}^{x^{2}}+s_{a}^{y^{2}}}$ $\alpha_{a}=\arctan\left(\frac{s_{a}^{y}}{s_{a}^{x}}\right)$$g_{b}=\sqrt{s_{b}^{x^{2}}+s_{b}^{y^{2}}}$ $\alpha_{b}=\arctan\left(\frac{s_{b}^{y}}{s_{b}^{x}}\right)$3: Calculate the relative intensity and orientation of the fused image and the single source image:$G^{\mathrm{ab}}=\{\begin{array}{c} \frac{g_{a}}{g_{b}},\;g_{a}<g_{b}\;\\ \frac{g_{b}}{g_{a}},\;g_{b}\leq g_{a} \end{array}$ $A^{\mathrm{ab}}=\frac{\left|\left|\alpha_{a}\;-\;\alpha_{b}\right|\;-\;\pi /2\right|}{\pi /2}$4: Calculate edge intensity preserving value and edge direction Preserving value:$Q_{g}^{\mathrm{ab}}=\frac{\Gamma_{g}K}{1\;+\;\exp\left[\kappa_{g}\left(G^{\mathrm{ab}}\;-\;\sigma_{g}\right)\right]}$    $Q_{\alpha }^{\mathrm{ab}}=\frac{\Gamma_{\alpha }K}{1\;+\;\exp\left[\kappa_{\alpha }\left(A^{\mathrm{ab}}\;-\;\sigma_{\alpha }\right)\right]}$5: Calculate edge information preserving value:$Q^{\mathrm{ab}}=Q_{g}^{\mathrm{ab}}\cdot Q_{\alpha }^{\mathrm{ab}}$6: Calculate edge intensity weight: $w_{a}={\left(g_{a}\right)}^{L}$7: Return $Q^{\mathrm{ab}}$ and $w_{a}$

**Algorithm 2** Compute Pull–Push Loss Value**Input:** visible image $I_{\mathrm{vis}}$; infrared image $I_{\mathrm{ir}}$; fusion result $I_{F}$**Output:** Pull–Push loss value $L_{\mathrm{Push}-\mathrm{Pull}\_\mathrm{SSIM}}$1: Calculate the weighted edge information preserving value of fused image and each image:$Q^{\mathrm{vis}},w_{\mathrm{vis}}=$ Compute Weighted Edge information Preserving Value ($I_{\mathrm{vis}}$,$I_{F}$)$Q^{\mathrm{ir}},w_{\mathrm{ir}}=$ Compute Weighted Edge information Preserving Value ($I_{\mathrm{ir}}$,$I_{F}$)2: Calculate normalized weighted edge information preserving values:

$Q^{\mathrm{vis}}=\frac{\sum Q^{\mathrm{vis}}{*w}_{\mathrm{vis}}}{\sum (w_{\mathrm{vis}}\;+\;w_{\mathrm{ir}})}{\;Q}^{\mathrm{ir}}=\frac{\sum Q^{\mathrm{ir}}{*w}_{\mathrm{ir}}}{\sum (w_{\mathrm{vis}}\;+\;w_{\mathrm{ir}})}$

3: Calculate the scale factor of Pull–Push loss:

$\alpha_{\mathrm{vis}},\alpha_{\mathrm{ir}}=\mathrm{Softmax}\left(\frac{Q^{\mathrm{vis}}}{Q^{\mathrm{vis}}\;+\;Q^{\mathrm{ir}}},\frac{Q^{\mathrm{ir}}}{Q^{\mathrm{vis}}\;+\;Q^{\mathrm{ir}}}\right)$

4: Calculate the structural similarity between the fused image and the single-source image:

${\mathrm{SSIM}}_{\mathrm{vis}}=\mathrm{MS}\_\mathrm{SSIM}\left(I_{\mathrm{vis}},I_{F}\right){\;\mathrm{SSIM}}_{\mathrm{ir}}=\mathrm{MS}\_\mathrm{SSIM}\left(I_{\mathrm{ir}},I_{F}\right)$

5: Calculate Pull–Push Loss Value:

$L_{\mathrm{Push}-\mathrm{Pull}\_\mathrm{SSIM}}=\alpha_{\mathrm{vis}}\left(1-{\mathrm{SSIM}}_{\mathrm{vis}}\right)+\alpha_{\mathrm{ir}}\left(1-{\mathrm{SSIM}}_{\mathrm{ir}}\right)$

6: Return $L_{\mathrm{Push}-\mathrm{Pull}\_\mathrm{SSIM}}$

## 4. Experiments

The purpose of this section is to demonstrate the superiority of our algorithm through experimental data. First, the module effectiveness of each design is demonstrated through several ablation studies. Based on this, the MSFAM method of this paper is compared with existing fusion algorithms to show that the proposed algorithm can achieve better performance in both qualitative and quantitative evaluation.

### 4.1. Implementation Details

All experiments were based on the Pytorch deep learning framework, implemented on a computer configured with Intel (R) Xeon (R) Silver 4110 CPU @ 2.10 GHz and RTX2080Ti GPU. If not otherwise specified, we trained the network using the Adam optimizer with a fixed 1 × 10^−4^ learning rate.

**Training**. Our training was divided into two phases. In the first stage, we connected the encoding part to the decoding part with separate infrared images or visible images to ensure feature extraction and reconstruction ability. We chose the FLIR data set for this phase, which contained 9676 visible images and 10,501 infrared images. We grayed visible images because of their multiple colors. We trained two epochs to ensure the completeness of the training. In the second stage, we added the attention feature fusion part between the pre-trained encoding and decoding parts and connected the Semantic Constraint network at the back of the whole model. The aligned pairs of visible and infrared images were fed into the network for the fusion training. We fused the TNO data set with the Road Scene data set, which contained a total of 273 image pairs, and we used 90% for training and 10% for fusion testing. During training, we ensured that the learning rate of the encoding part and Semantic Constraint network was 0; that is, we fixed the weights and guaranteed the original function. The learning rate of the decoding part was decayed to 50% of default to ensure that it had a certain degree of adaptive adjustment function. We trained 20 epochs and selected the best model for preservation. In addition, we trained the Semantic Constraint network to learn various image data distributions using visible and infrared images alone with fused images from some existing algorithms as inputs. The input images in the training phase were fixed at 320 × 256, with a batch size of 4 for the fusion model and 16 for the Semantic Constraint model. We used random horizontal and vertical flips for data enhancement to increase the scale of the data set.

**Inference**. We tested the model’s effectiveness using the remaining 10% of images from the fused TNO and Road Scene data set. We used the above two-stage fusion model without the Semantic Constraint module to achieve end-to-end fusion output of pairs of visible and infrared images. For testing, we used the original image size as input. For the output result, we normalized it to 0–255 according to the maximum and minimum data values to ensure the quality of visualization. For speed testing with other algorithms, we set the batch size to 1. We used six metrics—information entropy (EN), multi-scale institutional similarity (MS_SSIM), mutual information (MI), difference correlation (SCD), correlation coefficient (CC), and visual fidelity (VIF)—to quantitatively evaluate the quality of fused images. EN is used to characterize the richness of information contained in the fusion image; MS_SSIM, MI, SCD, and CC calculate the similarity or difference between the fusion result and the original image to show the retention degree of the original information in fused image; VIF realizes image quality evaluation based on natural scene statistics and human visual system. The detailed information and calculation of each metric can be found in related papers [42,43,44,45]. The larger the value of these metrics, the better the fusion results. Different from part of the original metrics that need a reference image for calculation, we use the mean value of two metrics, which are separately calculated by the fused result and two single-light images because there is no ideal reference. This is shown in the following formula.

(9) $$\mathrm{Metric}\left(F,V,I\right)=\frac{{\mathrm{Metric}}^{\prime }\left(F,V\right)+{\mathrm{Metric}}^{\prime }\left(F,I\right)}{2},$$

where F,V,I mean fusion result, visible image, and infrared image, respectively; Metric and ${\mathrm{Metric}}^{\prime }$ indicate the metrics in our paper and original metrics, respectively. Besides, we also provided three parameters for model complexity: Frame Per Second (FPS) and Giga Floating-point operations (GFlops) for time complexity and Parameters (Params) for spatial complexity. A larger FPS with fewer GFlops and Params indicates a more concise model.

### 4.2. Ablation Studies

#### 4.2.1. Effectiveness of Multi-Stage Training

To verify the effectiveness of our multi-stage training approach, we trained the same network using two existing training methods. To complete the experiments, minor modifications were made to our MSFAM model. For the “reconstruction training” mode, we only trained the encoding and decoding parts of the MSFAM model, similar to our first stage. For prediction, we used the hand-designed fusion module in NestFuse for visible and infrared feature fusion, whose manual attention fusion method is somewhat similar to ours and can be compared to some extent. For the “fusion training” mode, we trained our MSFAM network model using the traditional unsupervised training method without first-stage training. For the sake of comparison, we did not introduce the Pull–Push loss and Semantic Constraint modules, and only used the basic L1 loss and SSIM loss for training. The experimental results, shown in Table 1, demonstrate that the multi-stage training approach proposed in this paper could effectively improve the training effect of the model and ensure the final fused image quality from various aspects. The results show that the first stage enabled the network to obtain more accurate feature extraction and reconstruction capability through supervised training and reduced the difficulty of subsequent training. The second stage was fine-tuned based on the pre-trained model, which effectively ensured a better fusion ability for the dual-light feature compared with the manual method.

#### 4.2.2. Effectiveness of Attention Mechanisms

We then demonstrated the effectiveness of the designed fusion module based on the attention mechanism. For comparison, we replaced the module with 5 × 5 Conv layers to achieve the fusion of dual-light features. We also used the designed multi-stage approach for training. Table 2 shows that our designed attention fusion module could more effectively achieve the interaction and enhancement of dual-light features to meet the image fusion task to obtain a high-quality fusion result.

**Figure 6:**
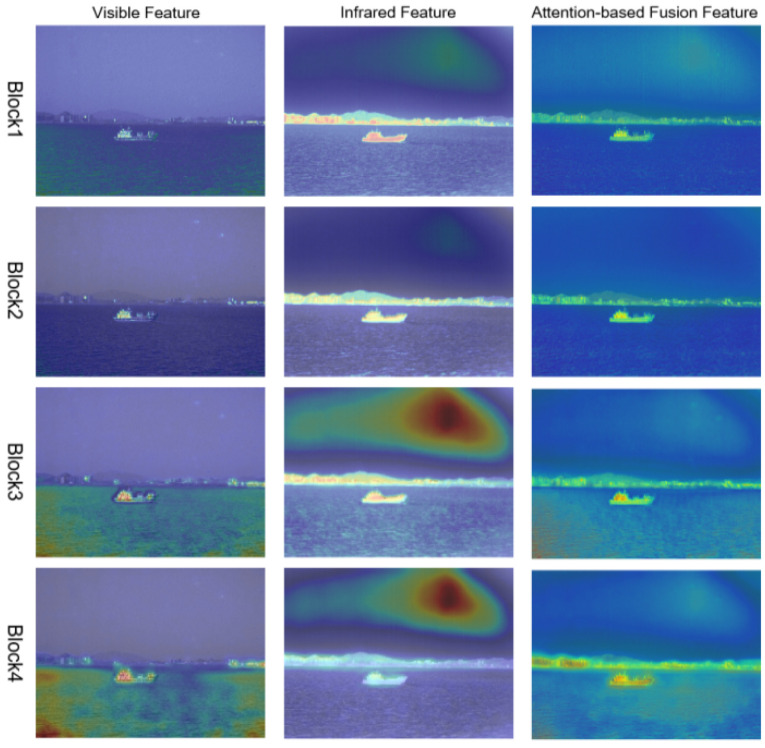
A visualization of the effectiveness of our attention fusion module.

We visualized the features of the attention fusion module, as shown in Figure 6, to show the effectiveness more intuitively. Each image shows the attention level of visible, infrared, and fused features to different regions, respectively. The single-light features directly output by the encoder module possessed their attention characteristics.

Similar to our analysis, the visible feature maps focused on the detailed information, such as the edge texture inside the ship and slight waves, while the infrared features focused more on the prominent targets with strong contrast, for the whole ship and the brightness background. We also found that the different layers had their attention regions, which also showed the effectiveness of feature reuse during reconstruction. Through the feature fusion enhancement process, it was obvious that the module achieved the effective fusion of dual-light features and achieved attention to the significant regions and the detailed edges simultaneously. In addition, we found that the infrared features paid a great degree of attention to the aerial spot region, which was unnecessary. Fortunately, it was reduced to a certain extent through the dual-light information interaction by the attention fusion module. This correction phenomenon indicated the effectiveness of the feature fusion module designed in this paper in another way.

#### 4.2.3. Effectiveness of Pull–Push Loss Function and Semantic Constraint Module

Based on the multi-stage attention fusion model, we compared the effect of introducing the Pull–Push loss function and Semantic Constraint module (or not introducing it) to demonstrate the effectiveness of the relevant improvements. Based on the pre-trained model that completes the first stage, we used four approaches to train the second-stage fusion. The experiments demonstrated that the introduction of the Pull–Push loss function and Semantic Constraint module alone could improve the image quality to some extent compared to the baseline, proving the effectiveness of the two types of improvements. Furthermore, the joint introduction enhanced the fusion effect more significantly. Table 3 shows that the introduction of constraint ideas from image content and deep semantic perspectives could effectively raise the training quality, and different components worked as a coherent module.

#### 4.2.4. Analysis on Factors of Loss

We further analyzed the effects of different loss factors on the fusion result. There are two major factors in our training stage, including the factor of Semantic Constraint loss $\alpha_{2}$ and the factor of Pull–Push loss β, so we ablate them in a two-stage method. We first keep a proper factor of Semantic Constraint loss unchanged, $\alpha_{2}$ = 1, and adjust the factor of Pull–Push loss β. Then, we maintain the factor of Pull–Push loss corresponding to the best result of above, i.e., β = 2, and change $\alpha_{2}$. The experimental results are shown in Table 4 and Table 5. Studies show that modifying both of the two factors can affect the final fusion effect to a certain extent. Finally, we choose $\alpha_{2}$ = 0.84 and β = 2 as the default by combining the results of multiple quality metrics.

### 4.3. Comparison with Recently Published Methods

We compared our models with existing state-of-the-art algorithms both in qualitative and quantitative aspects. We selected 10 methods in total for comparison, including three traditional algorithms (e.g., Hybrid_MSD [46], TIF [7], and NSCT_SR [6]), and seven deep learning models (e.g., DenseFuse [20], U2Fusion [15], SEDRFuse [21], PMGI [47], DualBranch [18], DIDFuse [19], and NestFuse [8]).

#### 4.3.1. Qualitative Comparison

**Figure 7:**
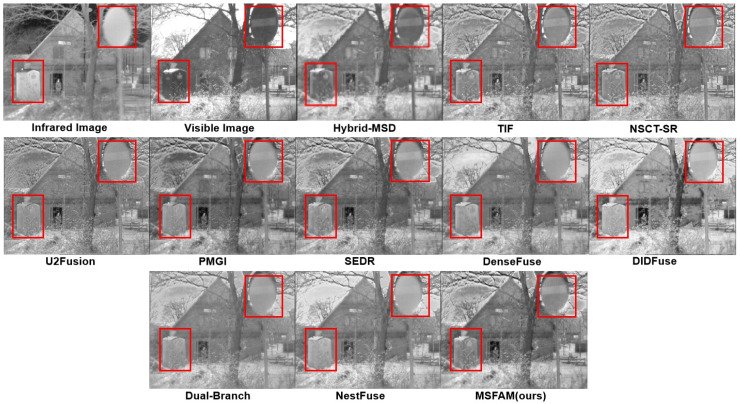
Experimental results of various methods on the “man in doorway” images. Red square represents for the areas that need to be focused.

**Figure 8:**
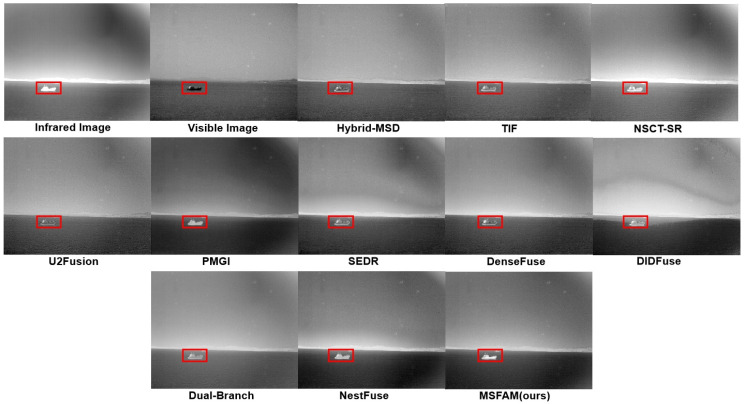
Experimental results of various methods on the “ship” images. Red square represents for the areas that need to be focused.

**Figure 9:**
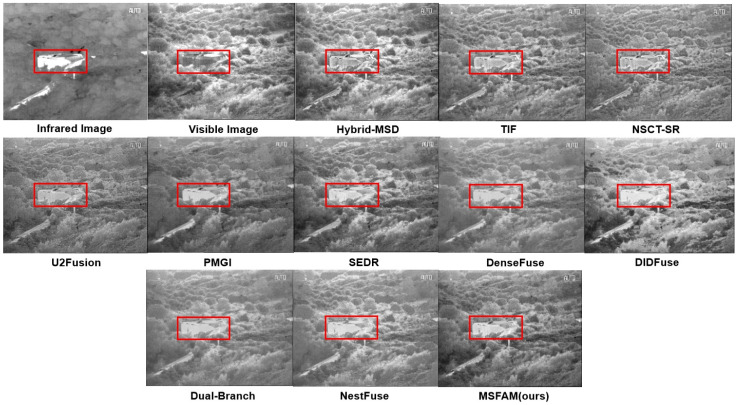
Experimental results of various methods on the “bunker” images. Red square represents for the areas that need to be focused.

Figure 7, Figure 8 and Figure 9 show the fusion results of each algorithm on part of the images. It can be clearly seen that compared with the existing algorithms, the proposed algorithm could ensure the contrast saliency of each object and balance the brightness difference between the target and the background. At the same time, the texture edge information of the original visible light was retained more completely, which could achieve the complement of the infrared detail information with a natural combination and without obvious artifacts. On the surface, our fusion result may not be significantly different from an existing algorithm in a single scene, such as Hybrid-MSD in Figure 7 and PMGI in Figure 9. However, it can be seen that MSFAM can output the most natural and beautiful fusion image in each of them. Thanks to the learnable attention fusion module, our model has a strong scene adaptability compared with others, which could be an effective improvement.

As for Figure 7, our algorithm better preserved the internal information of the signage, thanks to the multi-stage training approach we designed, which better realized feature extraction, fusion, and reconstruction. When there is a great pixel value difference of single object between two images, e.g., the ship in Figure 8, our model can effectively keep the high salient of target by adaptively discarding some low grayscale information. Besides, compared with several existing algorithms, our fusion result appears more natural because it solves the problem of floodlight in the sky area to a certain extent. In addition, as shown in Figure 9, the algorithm in this paper was able to highlight the infrared high-brightness targets such as houses adaptively, and it simultaneously was able to attenuate the background grayscale to a certain extent without losing texture information, realizing the distinction of the foreground and background automatically, owing to the attention mechanism in our feature fusion module.

#### 4.3.2. Quantitative Comparison

In Table 6, the quantitative evaluation metrics of different fusion algorithms are used to show the superiority of the model in this paper more objectively. Besides the six quality metrics mentioned above, the computational cost, including Parmas, GFlops, and FPS, was also counted in this paper to describe the complexity of each fusion algorithm. From the table, it can be seen that our MFSAM algorithm achieved the best results in three of the six quality metrics, i.e., EN, SCD, and CC. Compared with the existing algorithms, which only obtained good scores on a single metric or a few metrics, our method ranked among the top three in all metrics. Specifically, MSFAM surpassed its baseline, NestFuse, in all metrics thanks to our multi-stage training mode with improved loss functions. DIDFuse was second only to ours in term of quality metrics. It used a UNet-like model structure with a targeted training idea for visible and infrared image fusion task. It had a certain degree of similarity with ours, and its superiority also shows the effectiveness of our research direction. In terms of model complexity, although our model has larger Params and GFlops, it still guarantees a high inference speed based on its compact structure. The speed of proposed two-stage model was in the middle and upper level of the existing “reconstruction training” and “fusion training” modes, which was much higher than the traditional fusion algorithms. In summary, while ensuring a certain degree of real-time performance, the algorithm proposed in this paper could output high-quality visible and infrared fusion images with a certain improvement compared to the existing algorithms, achieving start-of-the-art performance of several quality metrics on published datasets.

### 4.4. Performance on a High-Level Task

Other than low-level image process tasks, the main purpose of image fusion is to improve the performance of subsequent high-level tasks, such as detection, tracking, or segmentation. As mentioned above, our fusion model achieved more effective information fusion and could improve the salience of the target and the detailed texture information at the same time. Thus, it can provide a more reliable judgment basis for the subsequent tasks and thus improve the effectiveness of the high-level perception result. To demonstrate the expressiveness of the fusion image obtained in this paper, we experimented with it on the object detection task.

Commonly used detection datasets, such as MS COCO and Pascal VOC, only cover visible images without paired infrared images. Thus, we experimented on the KAIST Multispectral Pedestrian Detection Benchmark [48], which consists of 95,000 color-thermal pair images with campus, road, and downtown scenarios in the day and light. Three categories (person, people, cyclist) were manually annotated, for a total of 103,128 dense annotations and 1182 unique pedestrians. The benchmark’s rich scenes and substances enabled us to fully verify the effectiveness of our algorithm in a variety of common cases.

For the detection algorithm, we chose the Generalized Focal Loss V2 framework [49]. This model replaced the specific value regression of the traditional detection method by fitting a generalized probability distribution, which is more suitable for location subtasks, especially in a fuzzy boundary and occlusion situation. This model deals better with the characteristics of the chosen dataset with considerable pedestrian occlusion and a vague outline in the infrared image.

For comparison, we compared our model with several existing fusion methods that performed well in qualitative or quantitative experiments. We chose the Hybrid_MSD method, SEDR for its good qualitative result, DIDFuse for its great qualitative metrics, and NestFuse because it is our baseline. Besides, we also used single visible and infrared images to show the effectiveness of general image fusion task. We trained each GFLV2 model with an ImageNet pretrained Resnet50 backbone for 24 epochs equally. The commonly used mAP index, calculated by precision and recall, was also chosen to describe the evaluation result. AP_S_, AP_M_, and AP_L_ were the values of small, medium, and large objects, respectively.

The evaluation measure values are shown in Table 7. The experiment results show that compared to the single visible or infrared image, the fusion ones obtained better performance on AP, AP_S_, and AP_M_, with comparable results of AP_L_, which indicates that our fusion result with rich salience and detail could also improve the performance of the detection task. Comparing with other fusion methods, the fusion result of MSFAM greatly improves the accuracy of the detection model. It can achieve better fusion effect in a wider range of scenes, so as to ensure the input quality of the detection model. Even without extra experiments, we believe that this conclusion will hold for other high-level perception tasks, like tracking or segmentation.

### 4.5. Generality and Limitation

Although our model was designed for the visible and infrared image fusion task, its two-stage training method could be extended to several multi-source image fusion applications to enhance training effect, such as multi-focus, multi-exposure, or even multi-band image fusion. Its generality makes it suitable to any encoder/decoder-like structure in a fusion task. However, the additional loss function in the second stage, i.e., Semantic Constraint loss and Pull–Push loss, needs to be modified or replaced to meet the characteristics of the corresponding task rather than a simple application.

Besides, our model is only applicable to fusion of paired gray visible and gray infrared images due to the requirements for consistency of input channel by the model structure and loss function. It is hard to be directly applied to color visible image with gray infrared one, i.e., three channels vs. one channel, which could also be an attention direction for our future exploration.

## 5. Conclusions

In this paper, we proposed a multi-stage visible and infrared image fusion network based on the attention mechanism, which can achieve a better performance in several fusion quality metrics. Unlike the existing single training mode, a two-stage joint training approach was proposed, which effectively reduced the difficulty of the model training and provided a new perspective on improving the image fusion effect. Accordingly, the Pull–Push loss function and Semantic Constraint module were designed for the characteristics of the fusion task, and their effectiveness was experimentally demonstrated. Our experiments demonstrated the importance of the training strategy and loss function design in the unsupervised fusion task, which can be a major research focus in the future.

## Figures and Tables

**Table 1:** Ablation study on the effectiveness of the multi-stage training approach compared with existing single phases. **Bold** represents for the best result in comparison.

Training Stage	Quality Metrics					
	EN	MSSIM	MI	SCD	CC	VIF
Rec	6.97	0.54	13.93	1.83	1.03	0.79
Fusion	6.97	0.54	13.93	1.82	1.04	0.93
Rec and Fusion	**7.04**	**0.55**	**14.07**	**1.84**	0.97	**0.95**

**Table 2:** Ablation study on the effectiveness of attention mechanisms in the feature fusion module. **Bold** represents for the best result in comparison.

Fusion Mode	Quality Metrics					
	EN	MSSIM	MI	SCD	CC	VIF
w/o attention	6.98	0.54	13.94	1.82	1.04	0.89
w/ attention	**7.04**	**0.55**	**14.07**	**1.84**	0.97	**0.94**

**Table 3:** Ablation study on the effectiveness of Pull–Push loss function and Semantic Constraint module (P. indicates Pull–Push loss function; C. indicates Semantic Constraint module). **Bold** represents for the best result in comparison. ↑ represents an improvement compared with the original algorithm, and ↓ is the opposite.

P.	C.	Quality Metrics					
		EN	MSSIM	MI	SCD	CC	VIF
✘	✘	7.04	0.55	14.07	1.83	0.97	0.94
✓	✘	7.07 ↑	0.54 ↓	14.13 ↑	1.87 ↑	1.05 ↑	0.98 ↑
✘	✓	7.08 ↑	0.54 ↓	14.07 -	1.86 ↑	1.04 ↑	0.94 -
✓	✓	**7.09** **↑↑**	**0.54** **↓**	**14.17** **↑↑**	**1.88** **↑↑**	1.05 ↑	**1.01** **↑↑**

**Table 4:** Ablation studies on changing loss factor β while maintaining $\alpha_{2}$ = 1. **Bold** represents for the best result in comparison.

Loss Factor		Quality Metrics					
$\alpha_{2}$	β	EN	MSSIM	MI	SCD	CC	VIF
1	1	7.01	0.52	13.97	1.71	1.01	0.99
1	2	**7.04**	**0.53**	**14.08**	1.66	**1.02**	**1.13**
1	3	7.03	0.51	14.05	1.64	1.00	1.10

**Table 5:** Ablation studies on changing loss factor $\alpha_{2}$ while maintaining β = 2. **Bold** represents for the best result in comparison.

Loss Factor		Quality Metrics					
$\alpha_{2}$	β	EN	MSSIM	MI	SCD	CC	VIF
0.5	2	6.98	0.54	14.12	1.73	1.03	0.96
0.84	2	**7.09**	**0.55**	**14.16**	**1.87**	**1.04**	1.10
1	2	7.04	0.53	14.08	1.66	1.02	1.13

**Table 6:** Comparison results on the TNO and RoadScene data with several recently published methods. **Bold** represents for the best result in comparison. ↑ indicates the larger value the better and **↓** means the opposite.

Methods	Quality Metrics						Model Complexity		
	EN (↑)	MSSIM (↑)	MI (↑)	SCD (↑)	CC (↑)	VIF (↑)	Params (↓)	GFlops (↓)	FPS (↑)
Hybrid_MSD	7.0089	0.5274	14.0005	1.6165	0.8805	0.9063	-	-	0.12
TIF	6.7003	0.5413	13.3833	1.6498	1.0028	0.8476	-	-	8.32
NSCT_SR	6.6012	0.5378	13.1853	1.6202	1.0105	0.7534	-	-	0.14
U2Fusion	7.0228	0.5235	14.0284	1.8064	1.0270	1.0041	0.6 M	108	50.25
PMGI	7.0597	0.5206	14.1022	1.7197	1.0121	0.9162	0.04 M	25	99.00
SEDR	7.0882	0.5216	14.1792	1.8507	1.0339	1.0019	3.4 M	407	4.78
DenseFuse	7.0172	0.5071	14.0173	1.6199	0.9141	0.7864	0.1 M	57	19.57
DIDFuse	7.0780	0.4862	**14.1988**	1.8068	0.9889	**1.2053**	0.3 M	21	53.05
DualBranch	6.5897	**0.5580**	13.1622	1.6675	1.0205	0.4519	0.5 M	11	163.93
NestFuse	7.0767	0.5186	14.1363	1.7088	0.9388	0.8903	2.7 M	95	22.88
MSFAM (Ours)	**7.0924**	0.5502	14.1675	**1.8775**	**1.0448**	1.1048	3.8 M	106	37.17

**Table 7:** GFLV2 evaluation result of different types of images on the KAIST Multispectral Pedestrian Detection Benchmark. **Bold** represents for the best result in comparison.

Image Type	Epoch	AP	AP_S_	AP_M_	AP_L_
Visible	24	0.756	0.673	0.754	0.819
Infrared		0.764	0.708	0.762	0.820
Hybrid_MSD		0.765	0.703	0.761	0.821
SEDR		0.764	0.694	0.758	0.823
DIDFuse		0.764	0.700	0.754	0.820
NestFuse		0.766	0.708	0.763	0.815
MSFAM		**0.768**	**0.710**	**0.764**	0.819
